# Two-Dimensional Visualization of the Three-Dimensional Planned Sacroiliac Screw Corridor with the Slice Fusion Method

**DOI:** 10.3390/jcm10020184

**Published:** 2021-01-06

**Authors:** Maximilian Kerschbaum, Siegmund Lang, Florian Baumann, Volker Alt, Michael Worlicek

**Affiliations:** Department of Trauma Surgery, Regensburg University Medical Center, Franz-Josef-Strauss-Allee 11, 93053 Regensburg, Germany; maximilian.kerschbaum@ukr.de (M.K.); siegmund.lang@ukr.de (S.L.); florian.baumann@ukr.de (F.B.); volker.alt@ukr.de (V.A.)

**Keywords:** SI instability, SI screws, posterior pelvic ring fracture, intraoperative fluoroscopy

## Abstract

Insertion of sacro-iliac (SI) screws for stabilization of the posterior pelvic ring without intraoperative navigation or three-dimensional imaging can be challenging. The aim of this study was to develop a simple method to visualize the ideal SI screw corridor, on lateral two-dimensional images, corresponding to the lateral fluoroscopic view, used intraoperatively while screw insertion, to prevent neurovascular injury. We used multiplanar reconstructions of pre- and postoperative computed tomography scans (CT) to determine the position of the SI corridor. Then, we processed the dataset into a lateral two-dimensional slice fusion image (SFI) matching head and tip of the screw. Comparison of the preoperative SFI planning and the screw position in the postoperative SFI showed reproducible results. In conclusion, the slice fusion method is a simple technique for translation of three-dimensional planned SI screw positioning into a two-dimensional strict lateral fluoroscopic-like view.

## 1. Introduction

Fractures of the pelvic ring are severe injuries, which occur with an incidence between 0.3 to 8.0% of all fractures [1]. Thereby, the posterior pelvic ring is mostly affected with sacral ala fractures, transforaminal sacral fractures or disruption of the sacroiliac (SI) joint [2]. During the last decades, approaches to the pelvis were developed from large incisions to minimally invasive techniques. A widely used, minimal invasive technique to stabilize the posterior pelvic ring is a sacroiliac screw fixation (SI screws) [3]. The value of minimal invasive surgery is rising, due to reduced soft tissue damage, reduction of surgical side infection, and cosmetic benefit. A recent study showed that the minimal invasive stabilization with SI screws shows the same functional results as the open technique, but with less blood loss [4]. This is in accordance to several studies, which demonstrated a good clinical outcome, with less infection rates and reduced blood loss after SI screw fixation [5,6,7]. Nowadays, there are several options for stabilization of the dorsal pelvic ring such as transiliacal internal fixator (TIFI), lumbopelvic fixation constructs, and the less invasive sacroiliac screw fixation [8]. Hereby, the osseous corridor of the first and/or second vertebral body is used for screw placement. This technique is limited by anatomical conditions. An osseous corridor with a width of <12 mm is considered critical, a width of <8 mm as impossible for trans-sacral implant positioning [9]. König et al. showed suitable S1 corridors in 63% of male and 66% of female patients, respectively, and S2 corridors in 87% of male and all female patients of their investigated collective [10]. In consequence, intraoperative visualization of ideal screw placement is challenging, especially in the case of sacral dysmorphisms, which can result in narrow or oblique osseous corridors [5,7,11,12,13,14,15,16,17,18]. This is in accordance with Tonetti et al., who described this technique by means of intraoperative fluoroscopy as successful, but very challenging due to the importance of exact x-ray positioning for inlet/outlet and strict lateral view [19]. Nevertheless, exact screw positioning is crucial to prevent neurovascular injuries [20,21,22]. Preoperative computed tomography (CT) scans are used to identify the possible osseous corridor, which allows ideal screw positioning. In cases where there is no intraoperative 3D navigation available, application of a three-dimensional planned screw position to an intraoperative, fluoroscopic two-dimensional image may be helpful.

The aim of the present investigation is to develop a simple method to visualize the perfect SI screw corridor on a preoperative CT scan, and transfer screw reference points of an ideal SI screw to lateral two-dimensional images, corresponding to the lateral fluoroscopic view used intraoperatively while screw insertion.

## 2. Methods

CT scans of patients treated with percutaneous screw fixation (PSF) of the posterior pelvic ring between 2018 and 2020 were analyzed. Inclusion criteria were PSF for a traumatic instability of the posterior pelvic ring, a complete set of images (pre- and postoperative CT scans), and age over 18 years. Exclusion criteria were missing data (e.g., no pre- and postoperative CT scan) or an imperfect SI screw position in the postoperative CT images.

Retrospectively, we processed the SFI based on the preoperative CT with an axial slice thickness of 1 mm in a bone kernel reconstruction. The DICOM (Digital Imaging and Communications in Medicine) datasets were further processed with the freeware software package OsiriX MD (Pixmeo, Bernex, Switzerland). After determination of the ideal screw position and identification of ideal entry point and ideal screw tip on SFI, we correlated the SFI based on the preoperative scan to the SFI based on the postoperative scan to verify transferability.

### 2.1. Determination of SI Screw Corridor

**Figure 1:**
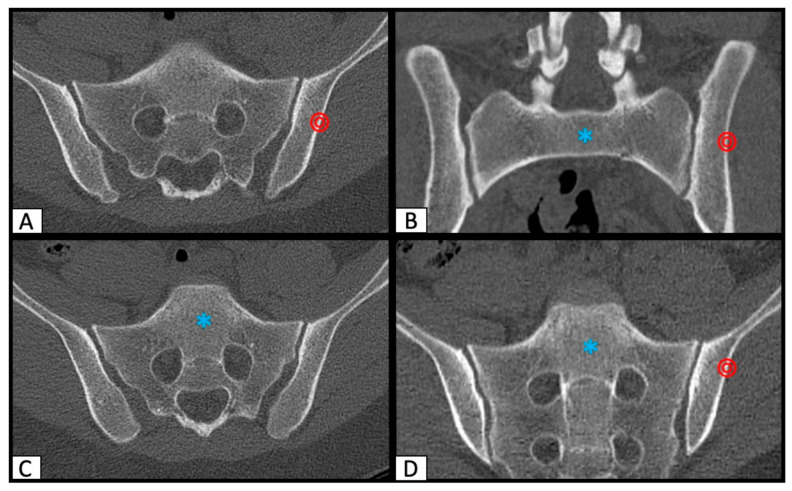
Determination of the perfect safe osseous corridor on axial (**A**), (**C**) as well as inlet (**B**) and outlet (**D**) orientated CT (computed tomography) scan slices. (**A**) shows the entrypoint (red circle), (**C**) the endpoint (blue asterix) for the SI (sacro-iliacal) screw in the axial view. (**B**) shows the planned position for the SI screw in inlet-, (**D**) in outlet orientated angulation.

Each CT scan was reformatted along the axis of the sacrum to have an exact reproducible, symmetric orientation using the multi-planar reconstruction function (MPR) of Osirix MD. Then, the perfect osseous corridor for a safe SI screw placement from the lateral ileum, through the ala sacralis into the body of the first and/or second sacral vertebrae was identified in axial and inlet/outlet orientated angulations (Figure 1).

Additionally, the actual screw position of the postoperative CT scans was determined in the axial, parasagittal, and coronal view. In the preoperative as well as in the postoperative CT slices, the entry point of the SI corridor and the end point of the corridor were marked by the setting of ROI (region of interest) points.

### 2.2. Image Processing with the Slice Fusion Method

**Figure 2:**
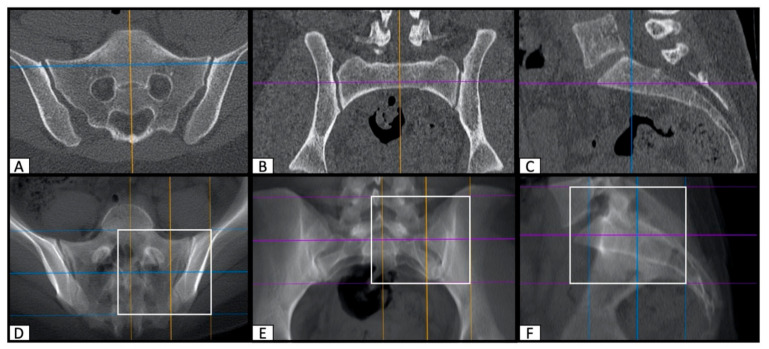
Symmetric orientation of the CT scans using the multi-planar reconstruction function of Osirix MD in axial and coronal orientations (**A**,**B**) to create an exact lateral view (**C**). Marking the region of interest (white box) and fusion of this area by means of the thick slab function (**D**–**F**) of OsiriX MD to create a two-dimensional exact lateral image of the sacrum (**F**).

After identification and marking of the SI corridor entry and end points, an image overlay of the CT slices was performed to visualize the marked ROI in a strictly lateral two-dimensional projection of the dorsal pelvis. First, exact lateral reconstructions of the pelvis were made and then a fusion of the relevant layers was performed. For this purpose, the thick slab function of OsiriX MD was applied in the multi-planar (MPR) reconstruction tool (Figure 2).

The resulting pre- and postoperative two-dimensional lateral projections of the SI screw corridor were compared with the intraoperative lateral fluoroscopic images.

### 2.3. Determination of SI Screw Corridor Entry- and Endpoint and Direction

**Figure 3:**
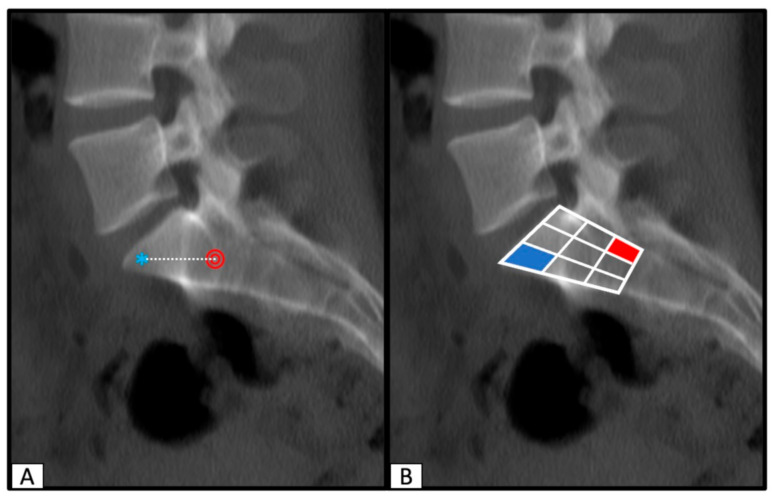
Processed image with the predicted entry- (red circle) and endpoint (blue asterix) of a planned SI screw (**A**). Division of the S1 vertebral body into nine segments to visualize the area for the entry- and endpoint of the SI screw and to predict the direction (**B**).

In order to visualize the position of the SI screw corridor on the strictly lateral view on the processed image, the S1 and/or S2 vertebral body was divided into three columns: an anterior, a central, and a posterior column. Those columns were then further divided into cranial, intermedium, and caudal segments. This resulted in a total of nine separate segments of the vertebrae body and enabled an accurate assessment of the entry-, endpoint, and direction of the SI screw corridor (Figure 3). Using this technique, pre-, intraoperative, and postoperative images were compared.

## 3. Results

In total, 30 SI corridors and placed SI screws were analyzed in this radiological investigation.

The identification of the perfect safe osseous corridor was reproducible in each preoperative CT scan (Figure 1). The fusion of CT slices by means of the thick slab function of OsiriX MD resulted in true lateral two-dimensional images, which were transferable to intraoperative fluoroscopy (Figure 2).

Comparing the preoperative predicted entry-, endpoint, and direction of the perfect osseous corridor to the actual postoperative position of every investigated SI screw resulted in almost identic SI screw positions. The nine-segment division of the vertebral bodies showed matching of the pre- and postoperative entry- and endpoints and made the direction of the screws comprehensible.

**Figure 4:**
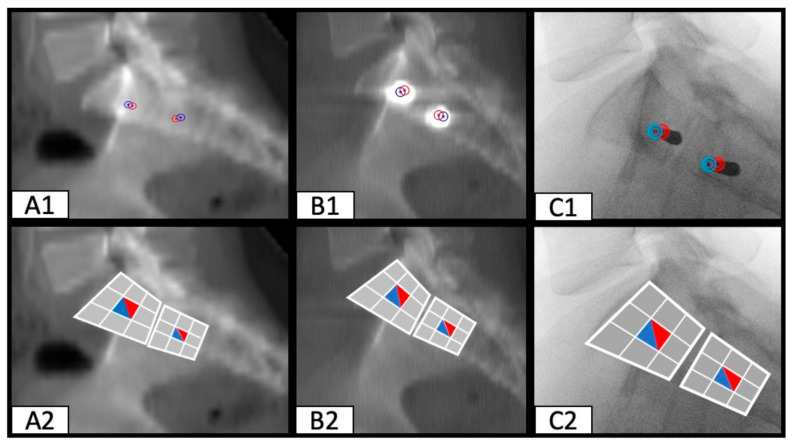
SI screw positioning in a patient with a straight osseous corridor (entry- and endpoint are close together). Predicted entry- (red circle) and endpoint (blue circle) of the perfect osseous corridor preoperatively (**A1**). Actual entry- (red circle) and endpoint (blue circle) of the inserted SI screw (**B1**). Intraoperative fluoroscopy image with an inserted k-wire, the red circle shows the entry point at the lateral iliac corticalis, the blue circle the tip of the k-wire (**C1**). Visualization of the predicted (**A2**), actual (**B2**), and intraoperative (**C2**) entry- and endpoints of the SI screws using the nine segment division of S1 and S2 vertebral bodies.

**Figure 5:**
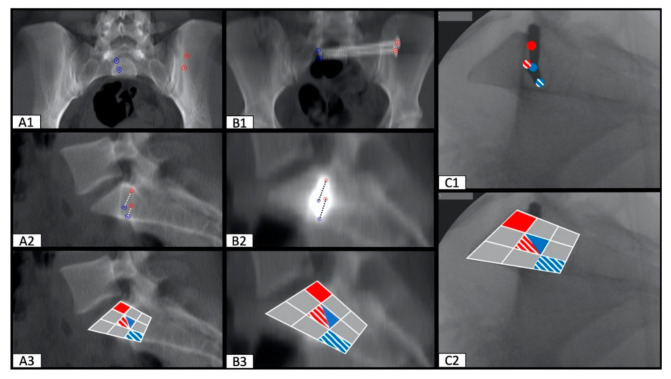
Positioning of two SI screws in a patient with a dysmorphic osseous corridor (entry- and endpoint diverge significantly). Predicted entry- (red circle) and endpoint (blue circle) of the perfect osseous corridor preoperatively (**A1**). Actual entry- (red circle) and endpoint (blue circle) of the inserted SI screw (**B1**). Intraoperative fluoroscopy image with an inserted k-wire, the red circle shows the entrypoint at the lateral iliac corticalis, the blue circle the tip of the k-wire (**C1**). Visualization of the predicted (**A2**,**A3**), actual (**B2**,**B3**) and intraoperative (**C2**) entry- and endpoint of the SI screw using the nine segment division of the S1 vertebral body.

Transferring these findings to the available intraoperative fluoroscopy images showed that the entry- and endpoint of the SI screw can be traced during surgery. These results apply to straight osseous corridors (Figure 4), as well as dysmorphic osseous corridors (Figure 5).

## 4. Discussion

The main finding of this study is, that the ideal position of SI screws in a safe osseous corridor can be planned preoperatively on CT scans and the entry-, endpoint, and direction of the SI screws can be transferred into a two-dimensional lateral, fluoroscopic-like image. This technique may be helpful for surgeons to re-identify the planned screw corridor intraoperatively.

Percutaneous SI screws insertion is a successful and minimal invasive technique for fixation of fractures of the posterior pelvic ring [23,24,25]. PSF has become the gold standard for the treatment of un-displaced posterior pelvic ring injuries [12,13,26,27,28,29]. Along with a rising number of pelvic fractures, there is a trend towards minimal invasive pelvic surgery. Pelvic surgeons have gained more and more experience with this technique. Two-dimensional fluoroscopy is the currently most common method for intraoperative imaging, with its advantages of low cost, widespread availability, and imaging in real time. Nevertheless, two-dimensional imaging of three-dimensional structures increases the risk of inaccuracy. This implies the risk of injury of neurovascular structures with instruments or screws. According to the literature, up to 24% of SI screws are mal-positioned, with risk of injury of sacral nerve roots, the descending lumbar nerve roots as well as the iliac vessels and their distal branches [12,30,31]. A reason for mal-positioning of SI screws is the anatomic variation of the safe osseous corridor [20,21,22,32]. Meanwhile, new techniques for accurate placement of SI screws are established. Intraoperative 3D-imaging or intraoperative image-based navigation can provide greater accuracy than conventional screw placement. The success rate of intraoperative navigation is reported with up to 96% of well-placed SI screws [33,34]. In addition to the higher accuracy, intraoperative navigation can reduce radiation exposure for especially young patients, the surgeon, and operating room personnel [35]. However, these techniques are associated with higher costs and of course, they are not available in every hospital.

The results of this study show that accurate planning of SI screw placement in preoperative CT scans, in combination with the slice fusion of sagittal CT images can provide a two-dimensional visualization of screw reference points (head and tip of the screw) in a lateral view, which may be reproducible intraoperatively by two-dimensional fluoroscopy. This technique might be helpful for the surgeon to identify the ideal position of an ideal SI screw, by using the fluoroscopy only in a strict lateral position. This could lead to a reduction of radiation exposure and might also reduce the operation time, as the surgeon does not need to change the position of the c-arm into inlet/outlet views several times to verify the direction of the k-wire. The technique also combines the advantages of three-dimensional planning on preoperative CT scans, with the widespread available and simple to use intraoperative fluoroscopy and a potential accuracy of intraoperative CT scans or 3D navigation.

This study has some limitations. First, the accuracy of the described technique is very sensitive to exact positioning of the c-arm. An exact, true- lateral view of the sacrum is crucial. This leads to a high subjective dependence of the performing surgeon.

Second, the evaluation was performed retrospectively on pre-, intra-, and postoperative CT scans and x-rays. Further clinical studies are needed to test this novel method with regard to intraoperative practicability and accuracy.

## 5. Conclusions

In conclusion, the slice fusion method is a simple technique for translation of three-dimensional planned SI screw positioning into a two-dimensional strict lateral view and might help intraoperatively to place SI screws using a lateral fluoroscopic image.

## Data Availability

Data sharing not applicable. No new data were created or analyzed in this study. Data sharing is not applicable to this article.
